# The role of perivascular adipose tissue-secreted adipocytokines in cardiovascular disease

**DOI:** 10.3389/fimmu.2023.1271051

**Published:** 2023-09-26

**Authors:** Meichao Cai, Dongsheng Zhao, Xiao Han, Shuang Han, Wenxin Zhang, Zhennan Zang, Chenchen Gai, Rong Rong, Tian Gao

**Affiliations:** ^1^ School of Pharmacy, Shandong University of Traditional Chinese Medicine, Jinan, China; ^2^ International Peace Maternity and Child Health Hospital, School of Medicine, Shanghai Jiao Tong University, Shanghai, China

**Keywords:** perivascular adipose tissue (PVAT), adipocytokines (adipokines), white adipose tissue (WAT), brown adipose tissue (BAT), cardiovascular disease

## Abstract

Perivascular adipose tissue and the vessel wall are connected through intricate bidirectional paracrine and vascular secretory signaling pathways. The secretion of inflammatory factors and oxidative products by the vessel wall in the diseased segment has the ability to influence the phenotype of perivascular adipocytes. Additionally, the secretion of adipokines by perivascular adipose tissue exacerbates the inflammatory response in the diseased vessel wall. Therefore, quantitative and qualitative studies of perivascular adipose tissue are of great value in the context of vascular inflammation and may provide a reference for the assessment of cardiovascular ischemic disease.

## Introduction

In recent years, high-calorie diets and low physical activity lifestyle habits have led to an increasing number of obese people. Being overweight is a major contributor to insulin resistance and cardiovascular disease. Insulin resistance and atherosclerotic disease are closely linked to obesity, especially abdominal obesity. In abdominal adiposity, the enlargement of visceral adipose tissue (VAT) is linked with larger adipocytes and impaired function of adipose tissue. This leads to elevated expression of inflammatory cytokines in adipocytes and macrophages infiltrating into adipose tissue, as well as the release of inflammatory cytokines by adipose tissue macrophages.

Perivascular adipose tissue (PVAT) is a special class of adipose tissue around blood vessels consisting of adipocytes, various immune cells and stromal cells. In healthy states, PVAT exerts a protective effect and reduces vasoconstriction. Evidence indicates that dysfunction of PVAT can cause inflammation in nearby arteries by releasing adipokines or inflammatory factors. These substances are then involved in the development of atherosclerosis, atherothrombosis, and plaque rupture. We argue that PVAT is important for the development of vascular disease; therefore, this paper reviews the paracrine function and pathological properties of PVAT.

## Perivascular adipose tissue

**Figure 1:**
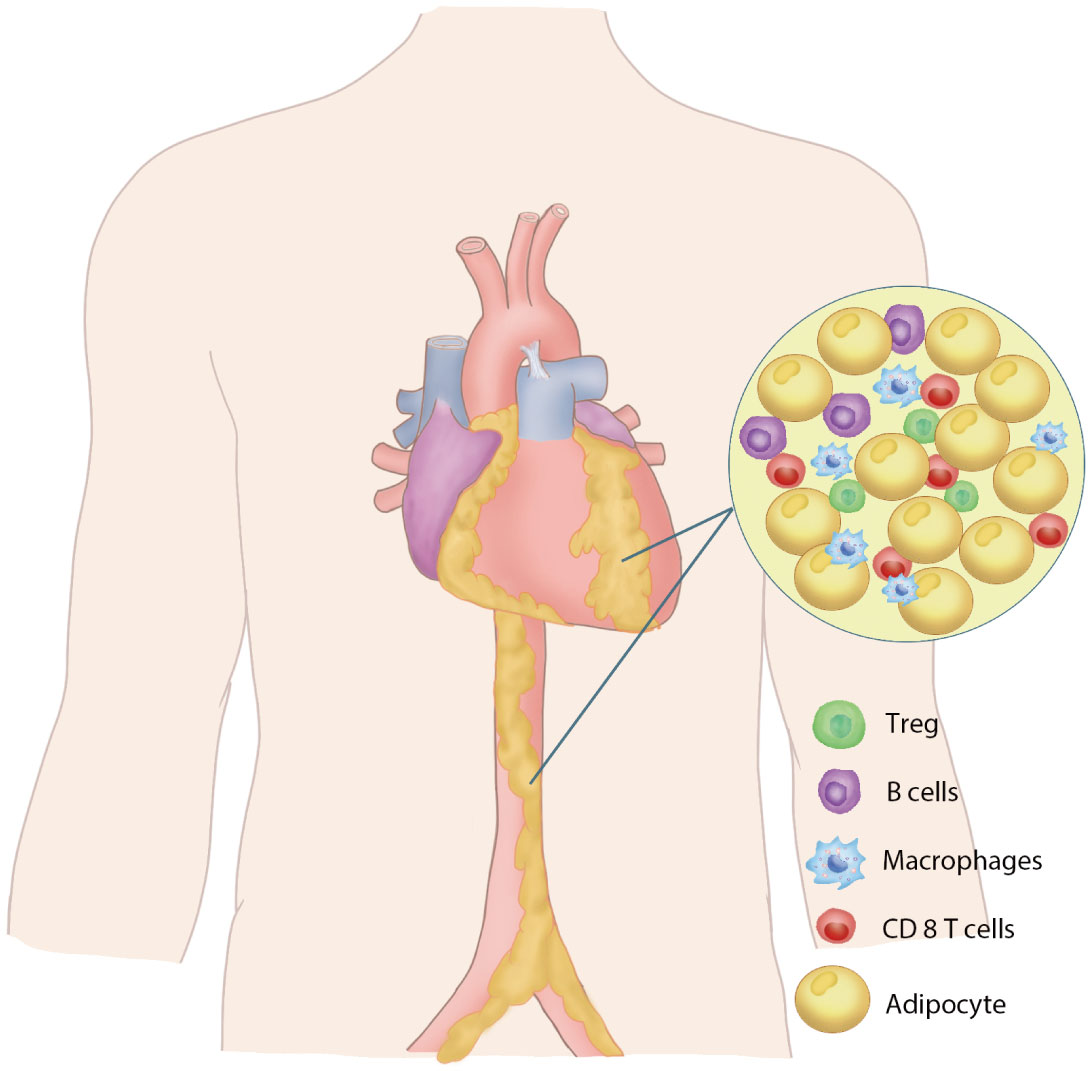
Perivascular adipose tissue. Schematic diagram of the composition of PVAT.

Perivascular adipose tissue is an adipose tissue close to the adventitial layer of blood vessels, which includes the periaortic fat and the specific fat depots of the major perivascular organs, pericardiac fat, and perirenal fat. PVAT is closely associated with the adventitial layer of the blood vessel wall in large blood vessels (1–3), while in small vessels and microvessels, PVAT is a component of the vessel wall without laminar structures (4, 5). PVAT consists primarily of adipocytes, preadipocytes, fibroblasts, macrophages, nerve cells and stromal vascular cells (6–8) (**Figure 1**). The perivascular adipocytes in the rodent thoracic aorta exhibit a similar phenotype to brown adipocytes, while white adipocytes are primarily present around the abdominal aorta. PVAT is different from other adipose tissues due to the interaction between brown adipose cells and white adipose cells and thus has different functional characteristics. In rodent studies, the morphology of adipocytes around the thoracic aorta of mice was mainly brown adipocytes, and the adipocytes around the abdominal aorta were mainly white adipocytes (9). Periaortic adipocytes in rats are significantly smaller than perimesenteric white adipocytes (10). Studies in humans have reported that the PVAT around the human coronary artery is white adipocytes (2), while the epicardial adipose tissue obtained from the origin of the human right coronary artery highly expresses the genes related to brown adipocytes (11).

There are clear histological differences between PVAT and typical subcutaneous WAT, with PVAT typically having smaller adipocyte volumes and less lipid storage and WAT showing lower levels of adipocyte-specific gene expression, indicating a significantly lower degree of differentiation than WAT (10, 12, 13). Coronary PVAT is considered to be more consistent with the histological manifestations and gene expression of WAT, but at the same time, animal experiments have confirmed that PRDM16 and UCP-1, the thermogenic markers of BAT, are expressed at higher levels in coronary PVAT than in subcutaneous adipose tissue (11). Compared with white adipose tissue, brown adipose tissue has smaller lipid droplets and is rich in mitochondria (14). Brown adipose tissue has a more vascular structure and denser innervation than white adipose tissue (15, 16). These differences may play an important role in the differences in the regulation of vascular function between the two types of adipose tissue. Thus, the complexity of perivascular adipose tissue underlies its regulation of vascular homeostasis and influence on cardiovascular disease.

## PVAT is a metabolically active endocrine organ

**Figure 2:**
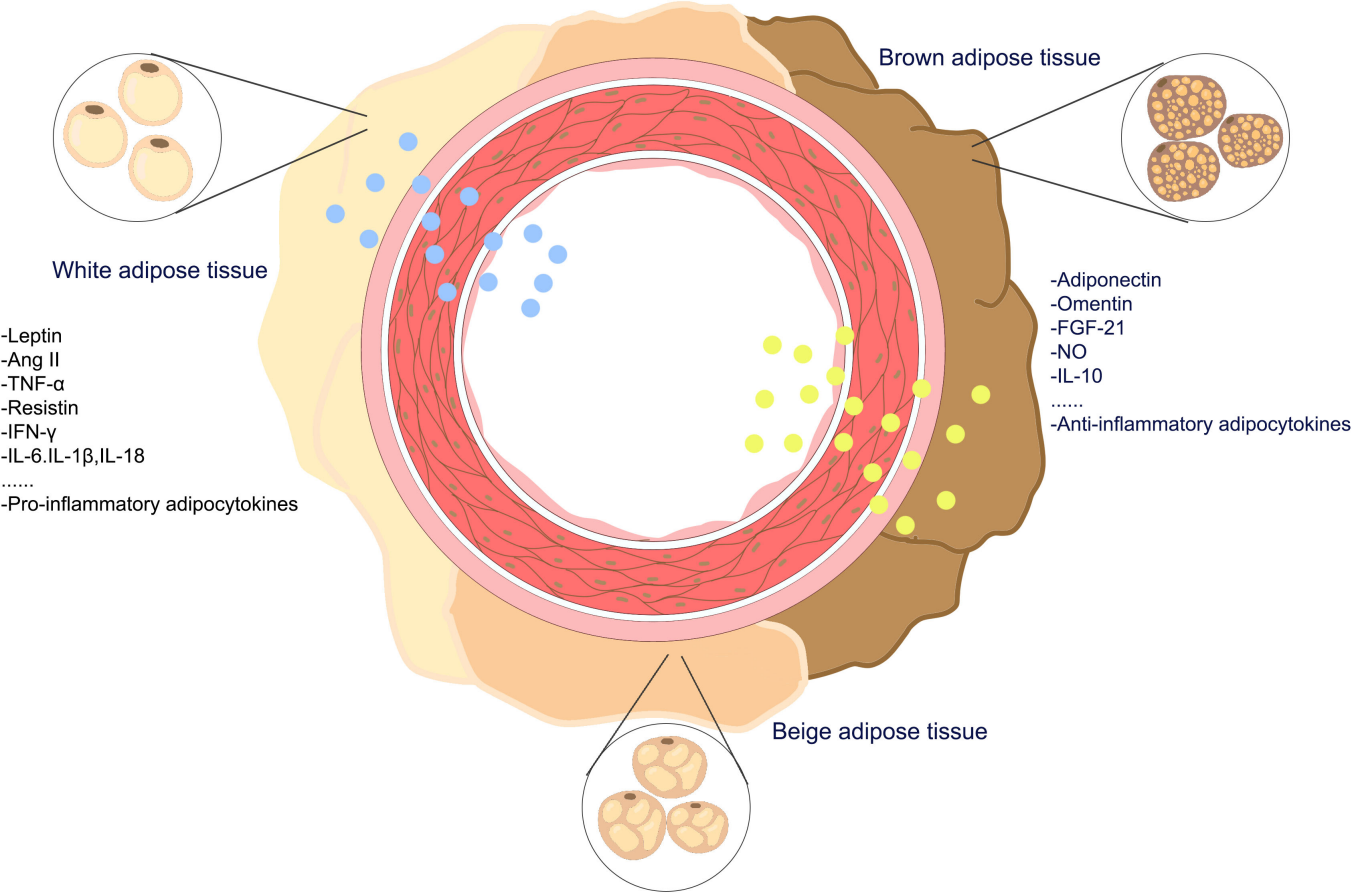
The adipokines of perivascular adipose tissueadipokines. PVAT is a complex adipose tissue composed of a variety of adipocytes, including white-brown adipocytes, beige adipocytes, and brown adipocytes, PVAT secretes pro-inflammatory and anti-inflammatory adipokines into the vasculature.

PVAT serves not only as connective tissue, providing support for arterial structures to protect them from damage by surrounding tissues (17, 18) but also as a crucial endocrine tissue involved in maintaining vascular homeostasis. PVAT secretes factors that can either promote vasoconstriction or vasodilation. Adipokines secreted by PVAT cells may contribute to the maintenance of vascular health and homeostasis under normal physiological conditions. Under the stimulation of pathological factors, PVAT can undergo phenotypic changes, cause dysfunction and inflammatory infiltration, and play an important role in the occurrence and development of vascular diseases. The representative chemokines produced by adipose tissue are adipokines such as adiponectin and leptin, along with other bioactive molecules such as miRNAs and proinflammatory cytokines such as interleukin (IL)-6 and tumor necrosis factor-α. PVAT controls the constriction of blood vessels and the restructuring of blood vessels by releasing active compounds such as adipokines, cytokines, and growth factors (**Figure 2**).

### Anti-inflammatory adipokines

#### Adiponectin

Adiponectin is a polypeptide specifically secreted by adipocytes, which was first identified in adipocytes and has high expression during adipocyte differentiation, and its monomer protein molecular weight is 30 kDa (19). The structure of the adiponectin monomer consists of four parts: the signal sequence at the amino end, the collagen domain, the highly variable nonhomologous sequence and the carboxyl end spherical domain (19). To date, three types of adiponectin receptors have been discovered based on their capability to bind to adiponectin. Adiponectin receptor 1 and 2 (AdipoR1 and AdipoR2), as well as T-cadherin (20, 21). AdipoR1 and AdipoR2 share a high level of structural similarity, with both proteins featuring seven transmembrane domains (20). T-cadherin is partially attached to the cell membrane via glycosylphosphatidylinositol, not transmembrane domains (21). Both the secretion and expression of adiponectin and the signal transduction of the adiponectin receptor play an important role in vascular homeostasis.

Adiponectin exhibits various beneficial effects, including promoting insulin sensitivity, preventing atherosclerosis, and reducing inflammation. It has a good inhibitory effect on various metabolic disorders, including type 2 diabetes, atherosclerosis, obesity, etc., and has a particularly prominent protective effect on the cardiovascular system (22–29). Epidemiological data and animal and cellular studies have yielded promising results on the cardioprotective effects of Adiponectin. There are extensive clinical epidemiological data suggesting a strong negative correlation between plasma levels of adiponectin and the occurrence rates of hypertension, left ventricular hypertrophy, and myocardial infarction (26, 30, 31). The findings indicate that the reduction in adiponectin levels could be a significant contributor to cardiovascular disease. Adiponectin affects the secretion of inflammatory factors via AMPK inhibition of the activated NF-kB signaling pathway, attenuating cardiac inflammation and high-fat diet-induced atherosclerosis. In pathological cardiomyocyte hypertrophy, Adiponectin inhibits cardiomyocyte hypertrophy by reducing markers that promote hypertrophy. In cardiac interstitial fibrosis, Adiponectin regulates cardiac interstitial fibrosis by affecting TGF-b and p-smad2/3 signaling pathways. A lot of research have shown that adiponectin contributes to glucose homeostasis and insulin sensitivity; however, adiponectin does not only protect blood vessels by improving insulin sensitivity. Some studies have provided evidence that low concentrations of adiponectin lead to a doubling of the risk of coronary heart disease compared to risk factors such as diabetes, hypertension and smoking (29). The results imply that adiponectin levels and cardiovascular disease risk are not influenced by blood glucose and lipid status, suggesting that adiponectin directly protects blood vessels rather than through insulin sensitivity and diabetes. High Adiponectin levels in the body tend to represent a healthier metabolic phenotype, however, Adiponectin also does not explain the beneficial phenotype in all healthy obese individuals. It has been shown that there is a paradox between Adiponectin and mortality in patients with cardiovascular disease, and that Adiponectin is positively associated with mortality in a variety of clinical conditions, including diabetes mellitus, which is related to confounding between lipocalin resistance and natriuretic peptides, and may be due to a combination of genetic, environmental, and dietary differences in different racial and regional populations.

#### Nitric oxide

PVAT affects the vascular response by increasing or inhibiting NO production (32–35). NO is synthesized by three NOS enzymes: neuronal (nNOS), inducible (iNOS) and endothelial (eNOS) (36). The various roles of NO in atherosclerosis are connected to the sources of NO. NO from eNOS and nNOS has antiatherosclerotic effects, while NO from iNOS can promote the occurrence of atherosclerosis. In nNOS^-/-^; ApoE^-/-^ mice, the aortic root and descending aorta showed greater atherosclerotic lesions and increased mortality (37). NO produced by nNOS in the cells of the central and peripheral nervous systems is involved in the regulation of blood pressure in the central nervous system as a neurotransmitter (38). Under normal physiological circumstances, there is no manifestation of iNOS within the vascular system. However, in instances of inflammation, sepsis, oxidative stress, and other pathological states, blood vessels exhibit iNOS. eNOS in PVAT directly produces and releases NO, affecting blood vessels (39). In addition, NO produced in PVAT positively regulates the release of adiponectin from PVAT (40). Therefore, improving NO secretion in PVAT is a promising target for cardiovascular disease therapy.

#### FGF21

FGF-21, belonging to the fibroblast growth factor family, fulfills a vital function by combating inflammation in both obese adipose tissue and heart tissue. FGF-21 exhibits important anti-inflammatory effects in obese adipose tissue, cardiac tissue and macrophages (41–45). FGF-21 gene expression is reduced in adipose tissue from patients with multivessel coronary artery disease associated with T2DM and enhanced in adipose tissue from patients undergoing cardiac surgery, suggesting a protective effect of FGF-21 against inflammation associated with cardiac surgery (46, 47). Hence, the anti-inflammatory impact of PVAT is advantageous in safeguarding against and treating cardiovascular ailments. On the other hand, the main role of FGF21 as an adipokine is to influence cellular lipid metabolism by modulating key transactivated receptors (such like ABCA1/ABCG1) and reducing macrophage-to-foam cell transformation (48, 49). Recent studies have shown that FGF21 can also act as a predictor of atherosclerosis by inhibiting mitochondrial fragmentation to reduce ROS production, and ultimately reducing NLRP3-mediated cellular death in endothelial cells to resist atherosclerosis (50, 51). The multifaceted functions of FGF21 in insulin sensitisation, lipid metabolism and inflammatory modulation collectively assist in its resistance to the progression of cardiovascular disease.

#### Omentin

One newly discovered adipocytokine called Omentin is found to be expressed in the omentum, epicardium, and perivascular adipose tissue (52, 53). Patients with heart failure have lower circulating levels of omentin, which attenuate myocardial ischemic injury by promoting mitochondrial biogenesis (54). Omentin exerts anti-inflammatory effects in smooth muscle cells by inhibiting TNF-α-induced VCAM-1 expression.Its mechanism may be associated with the inhibition of p38 and JNK activation (55). Omentin improves endothelial dysfunction in individuals with type 2 diabetes, and it also partially restores the loss of the anticontractile effect in PVAT. Additionally, Omentin treatment effectively enhances the pro-inflammatory and pro-oxidative phenotypes of PVAT (55). Omentin-1 was found to inhibit the expression of TGF-β1 in cardiomyocytes obtained from atrial fibrillation patients, reduce the activation of cardiac fibroblasts induced by TGF-β1, and reverse the endothelial-mesenchymal transition in HUVECs induced by TGF-β1 (56). Reduced levels of reticulin-1 in the circulation of patients with coronary artery disease (57, 58). Currently, there are fewer reports on the relevant role of Omentin in cardiovascular disease, mainly focusing on its ability to reduce vascular inflammation by affecting inflammatory factors such as TNFa and affecting vascular pressure through NOS-dependent pathways (59). Different research groups seem to have different views on the expression level of Omentin as a predictor in coronary heart disease (57, 60), which shows that there is still much unknown about its function as an adipokine in cardiovascular disease

### Pro-inflammatory adipokines

#### Leptin

Leptin is a non-glycosylated peptide hormone that is synthesized in adipocytes and has a molecular weight of 16 kDa. Leptin, a traditional proinflammatory adipokine, enhances the secretion of TNF-α, IL-6, and IL-12 by monocytes and increases the production of CC chemokine ligands (61, 62). It also promotes cell proliferation, reactive oxygen species (ROS) production, and the migratory response of monocytes (62). Furthermore, leptin is known to cause endothelial dysfunction by stimulating the production of C-reactive protein (CRP), cell adhesion molecules, and platelet tissue factor in endothelial cells (63). Increased levels of leptin have also been linked to obesity-related conditions such as myocardial infarction and stroke (64, 65). Leptin targets platelets, promotes platelet aggregation, stimulates platelet-dependent thrombosis and upregulates vascular adhesion molecules and prothrombotic tissue factor (66). The results indicate that leptin has the capacity to regulate cardiovascular disease. In a mouse model of atherosclerosis, Leptin intervenes in disease progression by affecting T-cell helper type 1 (Th1) response and promoting regulatory T-cell (Treg) function interferes with disease progression (67). In summary, Leptin’s main role is to influence vascular inflammation and regulate blood pressure in the body.

#### Resistin

Resistin, an Adipokine with Potent Proinflammatory Properties. PVAT-derived resistin plays a key role in diabetic vasculature via a pathway that involves the activation of AP-1 (68). In addition, resistin enhances the expression of chemokines MCP1, vascular cell adhesion molecule-1 (VCAM-1), and intercellular adhesion molecule-1 (ICAM-1) and promotes the release of inflammatory factors, such as interleukin and TNF-α, through the activation of the NF-κB signaling pathway, ultimately causing endothelial dysfunction (69–71). Current research on Resistin in cardiovascular disease is primarily related to hypertension. Increased levels of Resistin are present in both T2D or hypoxic conditions or obesity-induced hypertension, suggesting a positive correlation between Resistin and blood pressure (72, 73).

#### ANG II

ANG II functions primarily in cardiovascular disease to regulate blood pressure and vascular inflammation. ANG II, secreted by white and brown fat, regulates vascular tone and controls blood pressure. Mas receptor inhibition reduced the contractile and vasodilatory effects of exogenous ANG II on PVAT-denuded aorta. Ang II promotes inflammatory responses in PVAT and significantly downregulates oxidative phosphorylation in various regions of aortic PVAT, and the promotion of an adipocyte browning phenotype in aortic PVAT counteracts Ang II-induced inflammatory and oxidative effects (74). Ang II increases monocyte chemotactic protein-1 expression in the vascular wall and PVAT and Ang II-induced microvascular injury through modulation of the innate and adaptive immune responses (75). Thus, intervention in ANG II levels favors the regulation of blood pressure and vascular inflammation.

#### TNF-α and interleukin

TNF-α exerts both a proinflammatory effect and promotes the production of other adipokines, such as IL-6, leptin and resistin. Obesity promotes the secretion of large amounts of TNF-a by adipocytes and macrophages in the PVAT, and TNF-a promotes aortic intimal-medial thickening (76). TNF-α induces migration and proliferation of smooth muscle cells from the medial side of the vessel wall to the intima through upregulation of intercellular cell adhesion molecule-1 (ICAM-1), scavenger receptor class A and MCP-1 expression, ultimately leading to endothelial dysfunction (77, 78). TNF-α increases cytosis of lipoproteins between endothelial cells and macrophages, ultimately leading to retention of LDL in the vessel wall (79). IL-1β promotes immune cell recruitment and increases vascular permeability, Clinical use of canakinumab offers promise for targeting IL-1b for atherosclerosis treatment (80–82). IL-6 is another significant cytokine that plays a role in regulating the inflammatory response to PVAT. IL-6 acts directly on endothelial cells to increase superoxide production, leading to endothelial cell dysfunction (83). IL-6 affects vascular smooth muscle cell migration and induces vascular mitochondrial dysfunction in atherosclerosis (84). IL-18 modulates inflammation and plaque stability to promote atherosclerosis, but it is unclear whether IL-18 independently predicts atherosclerosis (85, 86).

PVAT-derived chemokines promote the recruitment of monocytes and T cells and further promote the production of chemokines and inflammatory factors (87). The function of cytokines produced by dysfunctional PVAT is multifaceted. The release of cytokines such as TNF-α, IL-6, and Il-1β, on the one hand, results in inflammation of adipose tissue and dysfunction of endothelial cells. However, IL-10 released by PVAT can enhance endothelial function by suppressing oxidative stress and augmenting the production of nitric oxide. Inflammatory and anti-inflammatory factors co-exist in vascular inflammation to maintain vascular homeostasis

#### miRNA

miRNAs, which are short, non-coding RNAs, can attach themselves to mRNAs, preventing their translation and subsequently impacting protein synthesis and various cellular processes. It has been shown that miRNAs in PVAT-secreted vesicles are associated with cardiovascular disease, for example, PVAT secretes extracellular vesicles containing miR-221-3p, which enhance the proliferation and migration of vascular smooth muscle cells, leading to vascular remodeling and dysfunction (88). Camila. et al. results suggest that miRNA-22 influences aortic reactivity in physiological circumstances and its deletion attenuates the loss of the NOS-mediated anticontractile effect of PVAT in obesity (89). According to reports, it has been stated that Perivascular adipose-derived exosomes decrease the formation of macrophage foam cells by upregulatingcholesterol efflux transporters ABCA1 and ABCG1 through miR-382-5p-and BMP4-PPARγ mediation (90). Although the current study confirmed that various miRNAs promote monocyte migration and pro-inflammatory. Although current studies haveconfirmedthat various miRNAs can promote monocyte migration and pro-inflammatory polarization and are involved in atherosclerosis, their specific roles in health and cardiovascular disease need to be further investigated (91).

## PVAT as a potential therapeutic target

PVAT is closely linked to cardiometabolic health and its white and brown adipocyte-driven switch may be a key factor in improved vascular health (92). Pan et al.found that the process of aging results in a reduction in the differentiation of brown fat. They compared young and aged perivascular adipose tissue-derived stromal cells using single-cell sequencing and discovered that aging results in a decline in PGC1α in these cells. This decline in PGC1α leads to reduced brown fat differentiation and encourages the deposition of collagen around blood vessels (93). The work of Adachid et al. suggests that inhibition of PVAT browning promotes perivascular macrophage inflammation exacerbating the vascular injury, therefore promoting PVAT browning would be beneficial in alleviating vascular inflammation and promoting vascular remodeling (94). In addition to the protective effects of BAT, two of Cao’s team’s works showed that BAT activation and WAT browning contribute to the progression of atherosclerosis by promoting lipolysis. One work showed that cold-induced BAT activation significantly increased plasma levels of small and medium low-density lipoprotein (LDL) residues by promoting lipolysis, which ultimately accelerated the development of atherosclerotic lesions in mice (95). Another work on Mirabegron, a clinical drug for the treatment of overactive bladder in humans, induces brown fat activation and white fat browning at the clinical dose. In contrast to the previously described brown fat activation and white fat browning followed by relief of atherosclerotic symptoms, oral administration of a clinical dose of mirabegron significantly accelerated atherosclerotic plaque growth and instability. The mechanism is that mirabegron promotes atherosclerotic plaque formation by increasing plasma levels of LDL cholesterol and very low-density cholesterol remnants through UCP1-promoted lipolysis (96). To summarize, the promotion of PVAT browning can have dual effects on atherosclerosis, both inhibiting and promoting it, depending on the circumstances. Therefore, how to target PVAT function to benefit the treatment of atherosclerosis requires further investigation.

## Conclusion

Perivascular adipose tissue plays a very important and complex role in the development of cardiovascular disease. It has been indicated by some evidence that the browning of perivascular adipose tissue could be advantageous for vascular well-being. For example, Age is an independent risk factor for vascular disease, the endothelial and brown adipogenic differentiation capacities of resident stromal cells in the PVAT decreases with age. Pan et al. found that resident stromal cells in the PVAT-induced vascular remodeling were ameliorated by overexpression of PGC1α, a key regulator of brown fat formation, ultimately alleviating age-related vascular dysfunction (93). Yusuke et al. found significant being of PVAT in lesions from patients with acute aortic coarctation. Furthermore, in a mouse model of intravascular injury, macrophages were found to accumulate in PVAT and promote adipose tissue remodeling, and being of PVAT could influence inflammation and vascular remodeling following vascular injury (94). There is also some evidence to suggest that brown adipose tissue-mediated lipolysis promotes atherosclerosis progression (95, 96). In conclusion, there is increasing evidence that the role of PVAT in CVD is multifaceted and that it may promote or resist CVD progression under different conditions. More research is required to evaluate if targeting PVAT function could be an innovative strategy for treating cardiovascular disease.

## Author contributions

MC: Writing – original draft. DZ: Writing – original draft. XH: Writing – original draft. SH: Writing – original draft. WZ: Writing – original draft. ZZ: Writing – original draft. CG: Writing – original draft. RR: Writing – original draft. TG: Writing – original draft, Funding acquisition, Writing – review & editing.
